# The Importance of Applying a Proper Splint in Fractures of the Patella

**Published:** 1898-11

**Authors:** J. S. Zvesper

**Affiliations:** Ammansville, Texas


For Texas Medical Journal.

### The Importance of Applying A Proper Splint in Fractures of the Patella.

BY J. S. ZVESPER, M. D., AMMANSVILLE, TEXAS.

That it is a matter of grave importance to apply a proper splint in a case of transverse fracture of the patella when everything else has been corrected is evident, and will be shown in the following report:

Mr. F. G., white; over 40 years old; a farmer; well built and healthy in every respect, sustained a transverse fracture of patella of the right knee while in danger of falling forward coming down from some steps, he attempted to recover himself by throwing his body backward. This caused violent contraction of the quadriceps muscles sufficient to fracture the patella.

He placed himself in the care of his physician, but finding, after a lapse of nine weeks, that his knee was no better, but rather worse, and utterly unable to perform any work necessitating the use of both legs, he applied to the writer for treatment.

I made a careful examination on July 25, and found the knee greatly swollen and painful to the touch; flexion impossible, so also extension; and the two fragments ununited as was clearly seen by a separation which was nearly three inches in extent.

An operation was proposed, to which patient willingly gave his consent. On July 27, 1898, the operation was undertaken, Dr. J. Adams administering the anaesthetic. The details of antisepsis and asepsis being closely adhered to, a longitudinal incision was made through the skin in the long axis of the limb, about the middle of the space between the fragments, from well above the middle of the fragment to well below the lower piece, through all the soft parts down to the seat of fracture.

Hemorrhage being controlled, the soft parts were retracted, periosteum remaining undisturbed, each of the two fragments was now drilled in one place. The adhesions and material of imperfect tendenous union were as far as was necessary and it was possible, removed. The jagged surfaces of the fragments cut square with a small saw through sound bone, and removed. The field of operation was now thoroughly irrigated, and every particle of loose tissue was washed off. Then a silver wire one-sixteenth of an inch in diameter was passed through the perforations,—the two fragments brought in apposition and the wire was tightened so as to hold the surfaces together. The ends were severed and stems hammered against the bone. A small drainage tube was inserted, the wound closed by interrupted sutures, dressed, and the limb placed in a splint.

The healing process was uninterrupted; in course of three weeks the leg was put in plaster of paris—the seat of operation being left exposed only, and the patient allowed to walk about, which he did without discomfort, except the weight of the plaster of paris, of course.

In two weeks the plaster was taken off. The leg could be flexed to certain extent without much difficulty; there was no more pain, and no swelling. Passive motion was advised, and after a week's time a marvelous change for the better occurred.

Patient is now up all the time and attending to his daily duties as before. I make this report thinking that it may be of interest and perhaps of benefit to some who may meet with a similar case for the first time.

